# Pre-Treatment and Post-Treatment I-131 Imaging in Differentiated Thyroid Carcinoma

**DOI:** 10.3390/jcm13071984

**Published:** 2024-03-29

**Authors:** Jasna Mihailović

**Affiliations:** 1 Department of Nuclear Medicine, Faculty of Medicine, University of Novi Sad, Hajduk Veljkova 3, 21000 Novi Sad, Serbia; jasna.mihailovic@mf.uns.ac.rs; Tel.: +381-63-526835; 2 Division of Nuclear Medicine, Oncology Institute of Vojvodine, Put dr Goldmana 4, 21204 Sremska Kamenica, Serbia

**Keywords:** differentiated thyroid carcinoma, radioiodine, whole-body imaging, planar, SPECT/CT

## Abstract

Radioiodine imaging in initial perioperative settings, after the total thyroidectomy, includes pre-treatment and post-treatment radioiodine imaging. While the benefit of post-treatment whole-body imaging (PT-WBI) is well established, the role of diagnostic whole-body imaging (dx WBI), prior to radioiodine (I-131) ablative or therapeutic doses, is controversial. Dx WBI has been abandoned in most nuclear medicine centers long ago. Planar low-dose dxWBI provides the volume of postoperative thyroid remnants, but it cannot detect occult metastatic foci in the neck. The modern integrated multimodality, i.e., SPECT/CT imaging, provides three dimensional images and accurate anatomic/metabolic data. This hybrid technology offers better spatial resolution but not better sensitivity. Dx WBI has low theranostic power because of the radioiodine indifference and low detection sensitivity for small-volume nodal disease in the neck. Since dx WBI cannot clarify the paratracheal cervical uptake, thyroid remnants may be easily misinterpreted as nodal disease, leading to a false N upstaging (from N0 stage to N1 stage) in DTC patients. Post-ablation I-131 imaging has a significant role in the initial staging of radioiodine-avid DTC and in the identification of non-radioiodine avid tumors. Additionally, SPECT/CT in the post-treatment setting provides more accurate initial TNM staging and better risk stratification of DTC patients. Post-treatment I-131 imaging is obligatory and must be performed in all DTC patients who receive radioiodine treatment.

## 1. Introduction

I-131 has been incorporated into the management of differentiated thyroid cancer (DTC) for many years. Following total thyroidectomy, the use of I-131 has been traditionally used as an important part of the initial DTC treatment algorithm. Over time, however, the role of postoperative I-131 ablation has changed. Since the recent data indicate that the I-131 ablation of thyroid remnants does not improve the outcome in low-risk DTC patients, postoperative ablation is recommended only for selective use in locally advanced and metastatic disease [1].

The postoperative radioiodine imaging of DTC includes postoperative (i.e., pre-treatment) diagnostic I-131, whole-body imaging (dx I-131 WBI), and post-I-131 treatment whole-body imaging (PT-WBI). While the role of post-treatment WBI is well established, the value of pre-treatment WBI remains uncertain.

## 2. Pre-Treatment Whole-Body Imaging

Pre-treatment I-131 WBI may be considered to determine the extent of postoperative thyroid remnants and to detect residual, previously unknown, regional or distant metastases. Remnant volume is influenced by the surgeon’s experience and technique [2]. After a total thyroidectomy is performed by an experienced and skilled surgeon, thyroid remnant uptake is usually lower than 2% [3].

SNMMI Practice Guideline for the therapy of thyroid disease with I-131 suggests that patients with large postoperative thyroid residuals are not able to receive subsequent radioiodine ablation safely due to the significant risk of radiation thyroiditis. Instead, they may be referred to thyroidectomy completion. In addition, this guideline claims that, “The preablation scan may alter staging when thyroid cancer is present, and hence change the activity of therapeutic I-131 to be administered” [4].

**Figure 1:**
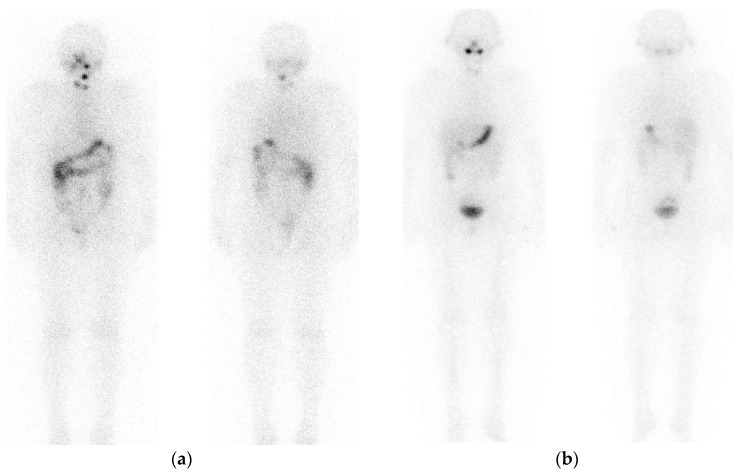
Stunning phenomenon in a DTC patient after total thyroidectomy. (**a**) Dx WBI planar images, anterior and posterior plane (after 111 MBq I-131), prior to I-131 therapy with 5.55 GBq, and (**b**) PT-WBI, planar images, anterior and posterior plane. High radioiodine uptake is seen in 3 foci in the thyroid bed, which is significantly reduced on the PT-WB images.

I-131 is the most utilized radiotracer for radioiodine imaging due to its low cost and wide availability. Dx WBI is usually performed using the low activity of I-131, 37–185 MBq (1–5 mCi), which has low sensitivity for the detection of occult disease [5]. In contrast, a higher amount of I-131 activity increases lesion detection and sensitivity [6,7,8,9] but may produce thyroid stunning. Stunning phenomenon decreases the uptake of the therapeutic dose [10,11,12,13], impairing the efficacy of radioiodine treatment [14,15,16,17]. Thyroid stunning is presented in Figure 1. 

The use of pre-treatment WBI is still a matter of debate. On the one hand, many authors advocate for this method, while on the other, numerous clinicians argue against it. Moreover, Van Nostrand et al. and van der Boom et al. claim that post-surgical dx WBI scans may detect additional information including cervical and distant metastases, which alter patients’ management. Their results obtained by pre-ablation scans changed clinical management in 4.2–29% of patients [18,19]. Many institutions can perform dxWBI with I-123 with a lower radiation-absorbed dose, low background activity, and better image quality [20,21,22]. Nevertheless, clinical management was altered in 5.7% of patients, changing radioiodine treatment in a two-step ablation by revealing large thyroid remnants [23]. Song et al., however, reported an alteration of therapeutic radioiodine activity in 49% of cases compared to the recommended I-131 activities based on surgical and histological reports only [24]. 

The literature data in DTC patients after a total or near-total thyroidectomy show the rate of successful remnant ablation regardless of whether they are treated with 1.1 GBq (30 mCi) or 3.7 GBq (100 mCi). Several long-term randomized trials have shown that remnant ablation with low activity versus high activity have equally effective clinical outcomes. Moreover, both the recurrence rate and mortality rate (or deaths) do not appear to be higher with the use of lower activities, compared to higher-dose-administered activities. However, short-term adverse effects are more frequent after the administration of 3.7 GBq versus 1.1 GBq. [1].

**Figure 2:**
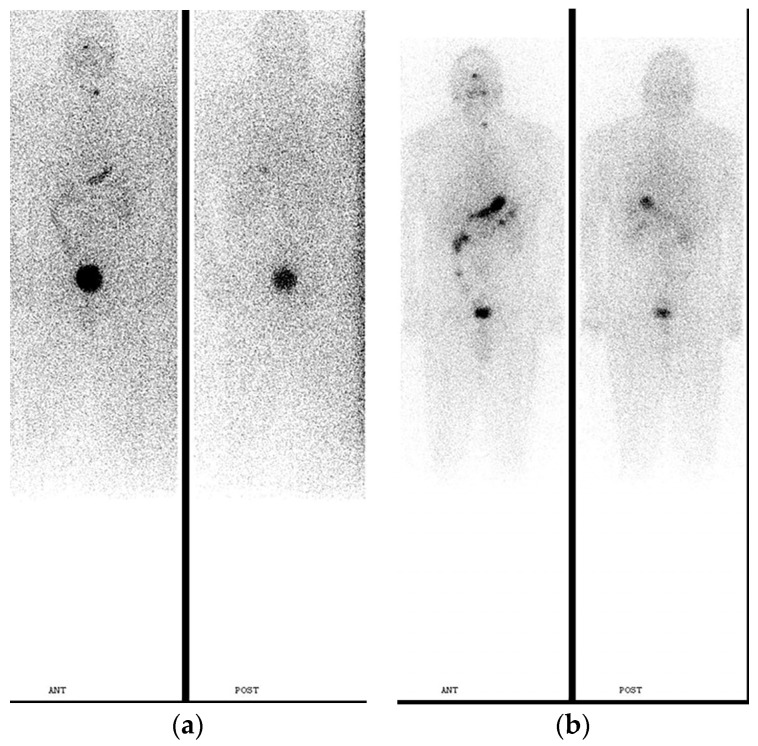

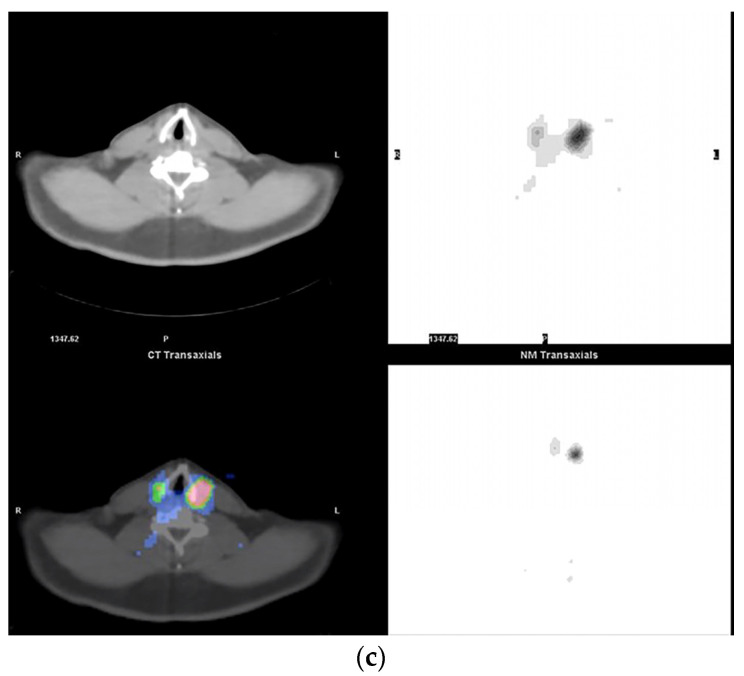
L.Y., a 42-year-old man, post total thyroidectomy and neck dissection, followed by I-131 ablation treatment with 1.1 GBq (30 mCi) I-131 for papillary thyroid carcinoma six months ago; initially staged pT3(m)N1Mx; TSH = 36.26 mU/L, Tgb = 4.5 ng/mL, TgbAb = negative. (**a**) Dx I-123 WBI, planar anterior and posterior images from the head to the knees and spot images of the neck were obtained following the oral administration of 74 MBq (2.0 mCi) I-123. There is a focal area of increased uptake in the region of the thyroid bed consistent with functioning residual thyroid tissue. There is no evidence of cervical or distant metastases. Normal physiologic activity is seen within the salivary glands, mucous membranes, gastrointestinal tract, and urinary system. (**b**) I-131 dx WBI planar and (**c**) dx SPECT/CT of the neck and chest with reconstructions in the axial, coronal, and sagittal planes were obtained 4 days following the oral administration of 185 MBq (5 mCi) I-131. The patient had received two IM injections of 0.9 mg rhTSH 24 and 48 h before receiving the tracer. The current scan demonstrates an area of increased focal uptake in the left thyroid bed and another area with less prominent radiotracer activity noted in the right thyroid bed; both foci without corresponding abnormality on CT, consistent with the thyroid gland remnant, left more than right. There is no evidence of functioning cervical or distant metastases.

I-124 has also been used for thyroid imaging utilizing PET/CT with high sensitivity and spatial resolution, further improving the identification of anatomic structures—residual thyroid tissue and metastatic lymph nodes. In addition, I-124 can be used for dosimetric studies [25]. PET/CT, however, is not as available as single-photon emission computed tomography/computed tomography (SPECT/CT), which provides functional (SPECT) and anatomical (CT) images of better quality and improved anatomic localization of lesions than planar imaging. SPECT/CT imaging is able to clearly demonstrate the exact localization of radioiodine uptake that was detected on planar WBI (Figure 2).

According to Avram et al., SPECT/CT is able to provide accurate localization of nodal foci on WBI [26]. In addition, Wong et al. claim that I-131 diagnostic SPECT/CT improves the planar diagnostic scan interpretation by detecting nodal metastases and clarifying ambiguous focal cervical uptake, subsequently altering prescribed therapeutic I-131 activity in 58% of patients [27].

Some authors suggest pre-ablation I-131 imaging with SPECT/CT to complete initial DTC staging prior to 131-I therapy [28,29,30]. Avram et al. analyzed pre-ablation 37 MBq (1mCi) I-131 planar and SPECT/CT imaging on a heterogeneous cohort of 320 patients initially stratified into low-, intermediate- and high-risk groups. The pre-ablation SPECT/CT scans detected regional metastases in 35% of patients and distant metastases in 8% of patients, leading to an alteration of clinical management in 4% of younger patients (<45 years) and 25% of older patients (age ≥ 45 years) [28]. Diagnostic I-131 WBI improved risk stratification with the detection of regional and distant metastases that altered patients’ management in 29.4% of patients [29]. Pre-ablation dxWBI was performed on 301 intermediate-risk DTC patients and detected functioning metastases in 10.6% of patients, upstaging into the high-risk category. In addition, patient management was modified in 8.3% of cases. Pre-ablation dxWBI using SPECT/CT detected significantly more nodal metastases than planar imaging (13.1% vs. 4.2%, *p* = 0.015) [30].

Recently, the SNMMI Procedure Standard/EANM Practice Guideline for Nuclear Medicine Evaluation and Therapy of Differentiated Thyroid Cancer has recommended SPECT/CT as the preferable imaging tool that should be used whenever available [31].

For more than a decade, the pre-ablation WBI, however, has been abandoned in many institutions worldwide. The nuclear medicine centers in the US prefer to perform the dx WBI routinely. In contrast, most European institutions discontinued pre-ablation imaging mainly due to the EANM-recommended guideline for radioiodine therapy of differentiated thyroid cancer. This guideline suggests that “In cases where RAIT clearly will be necessary, pre-therapeutic I-131 dxWBS or thyroid bed uptake measurement should be avoided because their results will not modify the indication for RAIT and these procedures may induce stunning” [32]. In addition, the British Thyroid Association’s guideline for the management of thyroid cancer suggests “A pre-ablation scan is not indicated routinely if the patient has had optimal surgery. If there is any doubt over completeness of surgery or radiological evidence of a large remnant, further resection should be discussed before proceeding to radioiodine remnant ablation” [33]. The ATA 2015 guideline offers a weak recommendation: “Pre-therapy scans and/or measurement of thyroid bed uptake may be useful when the extent of the thyroid remnant cannot be accurately ascertained from the surgical report or neck ultrasonography, or when the results would alter either the decision to treat or the activity of RAI that is administered” [1]. The last version of the NCCN Guideline (Version 4.2023) recommends pre-treatment radioiodine imaging as “may be considered”. With an aim to minimize stunning, this guideline suggests activity of 74–148 MBq (2–4 mCi) for Iodine-123 dx WBI or low activity of 37–74 MBq (1–2 mCi) for I-131 dxWBI [34].

Some authors advocate against diagnostic radioiodine imaging. They propose several scenarios when pre-ablation dxWBI can be omitted: (a) if small thyroid remnants, with uptake of less than 2%, remain after the surgery which was performed by well experienced surgeons; (b) if a thyroidectomized patient is routinely scheduled for radioiodine ablation regardless of the diagnostic imaging results; (c) if alternative diagnostic methods are used, such as ultrasound, for the determination of the extent of thyroid remnants or chest X-rays for the detection of lung metastases. Additional factors against pre-ablation imaging include patients’ inconvenience and the high cost of scanning [3,35,36]. In another study, empiric treatment has been found to be more cost-effective than management including diagnostic WBS with estimated savings of USD 13,750,000 each year in the US [37].

In addition, there are several factors that influence the results of dxWBI. Due to genomic mutations, some differentiated thyroid carcinomas show different levels of radioiodine avidity. Diminished theranostic power for radioiodine imaging and response to radioiodine therapy is characterized as “radioactive iodine indifference” [38,39]. 

Furthermore, different preparation protocols of pre-treatment imaging (using thyroid hormone withdrawal or rhTSH stimulation) have an impact on the sensitivity for lesion detection. Pre-treatment rhTSH-stimulated dxWBI has failed to detect remnants and/or thyroid bed tumor tissue in 16% and metastatic disease in 24% of cases if compared to dxWBI obtained after the thyroid withdrawal method [40,41,42].

The EANM guideline for the radioiodine therapy of differentiated thyroid cancer recommends that the dxWBI should be avoided in patients in whom radioiodine therapy is clearly necessary, since it will not modify the indication for radioiodine treatment and might cause thyroid stunning [31]. Similar to the EANM guideline’s recommendation [31], the recently published ETA (European Thyroid Association) Consensus statement advocates against pre-ablation imaging. Recommendation 7 suggests that “whenever a decision to perform postoperative RAI therapy needs to be taken, a diagnostic scan is not routinely required” [43].

Seza et al. raised concern regarding the pre-treatment WBI interpretation of the radioiodine uptake in the neck as “very important but not a well-recognized clinical problem”. The valid and accurate distinction between postoperative thyroid residuals and cervical metastases is crucial for the initial N staging in DTC patients. Hybrid SPECT/CT imaging, however, besides better specificity, does not offer better sensitivity over planar imaging. The pre-treatment diagnostic radioiodine imaging is recommended only selectively in intermediate-risk DTC patients [38]. An additional challenge in SPECT/CT imaging exists in critical post-surgical thyroid remnants left around anatomical structures such as Berry’s ligament or Zuckerkandl tubercle. These focal remnants can be misinterpreted for cervical lymph node metastases and result in the false upstaging of patients to stage N1 from stage N0 [2,44].

Dx WBI has a low theranostic power due to radioiodine indifference and low detection sensitivity for small-volume cervical nodal disease. Pre-ablation WBI cannot differentiate between cervical metastatic residual disease and benign thyroid remnants.

The current clinical practice for DTC treatment usually involves the administration of fixed or empiric activities of I-131 between 3.7 and 7.4 GBq. According to the recent SNMMI Procedure Standard/EANM Practice Guideline, the suggested algorithm for risk-adapted I-131 therapy includes remnant ablation in low-risk patients performed with 1.11–1.85 GBq (30–50 mCi), adjuvant treatment performed with 1.85–3.7 GBq (50–100 mCi), while treatment of a known disease performed with 3.7–5.6 GBq (100–150 mCi) for small-volume locoregional disease and 5.6–7.4 GBq (150–200 mCi) for the treatment of advanced locoregional disease and/or small-volume distant metastatic disease. In patients with diffuse distant metastatic disease, higher I-131 activities are recommended, ≥7.4 GBq (≥200 mCi), which are determined by dosimetric calculations [31].

The standard activity approach, so called empiric or fixed method approach, fails to personalize the radioiodine therapy. Specifically, this approach may result in overtreatment—the patient’s higher radiation exposure, or undertreatment—and administration of insufficient initial I-131 activity may result in a less efficient I-131 treatment outcome. On the contrary, dosimetry plays an important role in the radioiodine treatment algorithm and is based on dosimetric studies and calculations. This approach involves: (a) the blood or bone marrow dosimetry method and (b) lesion dosimetry method. The blood/bone marrow-based method is more frequently performed and includes a calculation of maximum-tolerated dose (MTD) with the blood as the critical organ in order to avoid severe radiation effects on the bone marrow. It is based on the clinical observation that there is no myelotoxicity if patient’s blood-absorbed dose is <2 Gy [31,45]. This method includes radiation count measurements of serial blood samples and uptake probe measurements of the patient’s whole-body activity during the 4 or more-day examination after the administration of I-131 tracer. Blood dosimetry on the one side permits the administration of I-131 activities ≥5.55 (150 mCi) or even of 12.95–18.5 GBq (350–500 mCi) to avoid myelotoxicity, and on the other side, to identify those who cannot receive even 5.55 GBq (150 mCi) safely [46]. 

Lesion-based dosimetry determines the radiation-absorbed dose that would be delivered to a lesion, thyroid remnant (≥300 Gy) or metastatic disease (≥80 Gy), minimizing the patient’s risk. Some centers, however, combine lesion and blood dosimetry methods, while an accurate calculation of the lesion volume of metastatic and remnant tissue remnants remains one of the main problems in this individualized treatment approach [45]. In order to determine the activity of I-131 that will not exceed 2 Gy of blood-absorbed dose, the EANM Dosimetry Committee has published guidelines for the standard procedure in dosimetry [47].

Currently, there are no published literature data that show advantages of a dosimetric approach over an empiric approach in the treatment of loco-regional or metastatic disease. Furthermore, according to the ATA 2015 Guidelines, “no recommendation can be made about the superiority of one method of RAI administration over another (empiric high activity vs. blood and/or body dosimetry vs. lesional dosimetry)” [1].

Although the method complexity and patient’s inconvenience are disadvantages of a dosimetric study, this approach improves the management of metastatic DTC patients with providing the optimal individual treatment.

## 3. Post-Treatment Whole-Body Imaging

According to the NCCN Guideline (Version 4.2023), post-treatment WBI should be performed in all patients after radioiodine therapy [34]. PT-WBI is usually performed within one week after I-131 therapy with an aim to assess the extent of radiodine-avid disease and to complete the initial staging of DTC patients. Additionally, it also discloses radioiodine non-avid tumors [38]. In comparison to other scanning modalities, post-treatment WBI may be considered as the gold-standard imaging technique.

**Figure 3:**
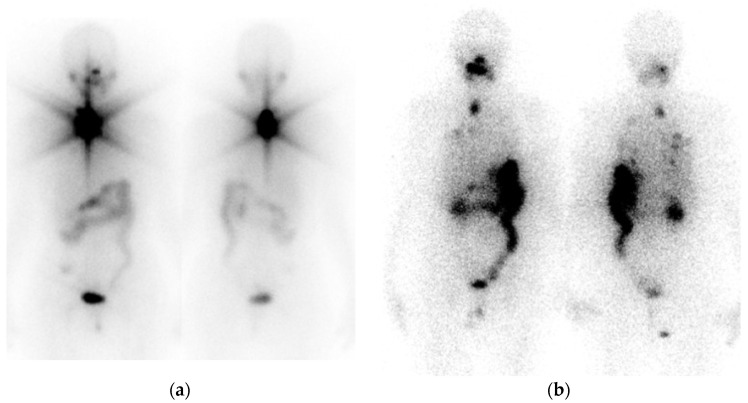
G.G., a 70-year-old woman, after total thyroidectomy, papillary thyroid carcinoma, initially staged pT1aN0Mx; TSH = 125 mU/L; Tgb (ICMA) = 3028 ng/mL; TgbAb = negative. (**a**) dxWBI, planar study, anterior and posterior whole-body images and spot images of the neck were obtained 24 and 48 h after oral administration of 37 MBq (1mCi) of I-131. Anterior and posterior whole-body images show a focus on increased activity in the region of the thyroid bed, consistent with thyroid remnants. There is an area of slightly increased activity in the chest, in the region of the right hilum, suspicious for metastatic disease from thyroid carcinoma; (**b**) PT-WBS after administration of 9.1 GBq (246.8 mCi) I-131 anterior and posterior images from the head to the knees and spot images of the neck demonstrate increased uptake in the region of the thyroid bed, corresponding to residual thyroid tissue. There is a focal area of markedly increased activity in the chest, in the region of the right hilum, compatible with metastasis. Further evaluation shows a small focus of slightly increased activity in the region of the left lower lobe, suspicious for metastasis.

**Figure 4:**
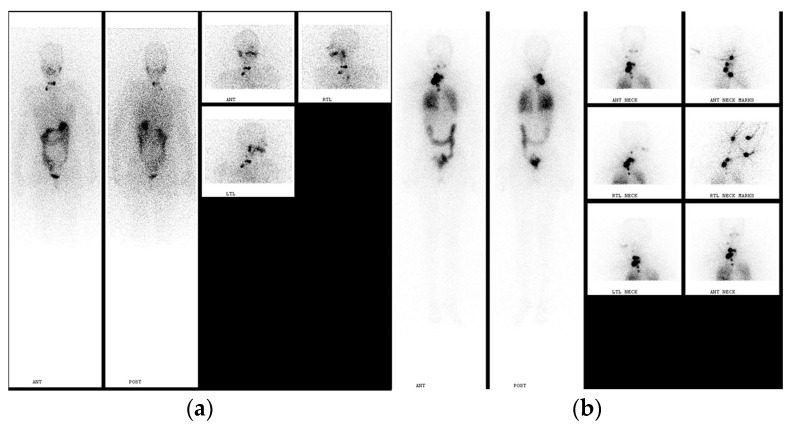
G.T., a 19-year-old woman, after total thyroidectomy and modified neck dissection, papillary thyroid carcinoma, initially staged pT2 (m)N1Mx. (**a**) Dx I-123 WBI, anterior and posterior images from head to knees, and spot images of the neck were obtained following the oral administration of 81.4 MBq (2.2 mCi) I-123. Additional focus is located at the base of the neck on the right, likely consistent with the right cervical lymph node metastasis; Tgb (RIA) = 869 ng/mL; TSH = 100 mU/L; TgbAb = negative. The I-123 scan demonstrates 2 foci of increased uptake in the region of the thyroid bed corresponding to functioning residual thyroid tissue. Two additional foci are seen inferior to the thyroid bed; the first focus is located at the base of the neck on the right and the second in the midline chest. The first focus corresponds to right cervical lymph node metastasis, while the focus in the midline chest likely represents esophageal uptake. (**b**) PT-WBI after administration of 9.2 GBq (249 mCi) I-131, anterior and posterior images from the head to the knees, and spot images of the neck demonstrate increased uptake in the region of the thyroid bed corresponding to the residual thyroid tissue. A focal area of uptake is again seen in the right neck. There are 2 new foci within the midline chest corresponding to the cervical and paratracheal lymph node metastases. There is intense diffuse uptake throughout the lungs, which is bilaterally consistent with diffuse bilateral pulmonary metastases.

Post-ablation imaging shows higher sensitivity in comparison to pre-ablation scanning. PT-WBI may disclose the presence of unsuspected metastatic lesions in 10–26% of high-risk patients [48,49], and much less in low- and intermediate-risk DTC patients (in ~2% of cases), leading to re-classification of DTC staging [50]. PT-WBS images that show new metastatic foci, which were not seen on dxWBI, are shown in Figure 3 and Figure 4. 

Appropriate timing for PT-WBI is a challenging issue. The literature data show different results if comparing early and late PT-WB imaging [51,52,53,54]. Metastatic lesions, including 28% of lymph node metastases, 17% of lung metastases, and 16% of bone metastases, were seen only on early scans (performed on the 3rd–6th day), in contrast to delayed scans performed on the 10th–11th day [51]. A similar study was performed by Salvatori et al. who compared early post-treatment scans performed on the 3rd day with delayed WBI performed on the 7th day. They obtained discordant images between early and delayed scans in 19.5% of patients; in total, 7.5% of lesions (including nodal and distant metastases) were detected only on early scans, while 12% of lesions (including thyroid remnants, nodal or distant metastases) were seen only on the late scans [52]. Concordantly, Chong et al. claim that late PT-WB imaging is favorable over early PT-WBI since it provides better contrast resolution as a result of radioiodine body clearance. They detected lung and bone metastases in 22% and 33% of patients, respectively, only on late PT-WBI [53]. Similar results were demonstrated in another study carried out by Kodani et al., who reported lung metastases in 29% and bone lesions in 20% of patients, detected only at late PT-WBS performed on the 7th–9th day [54].

**Figure 5:**
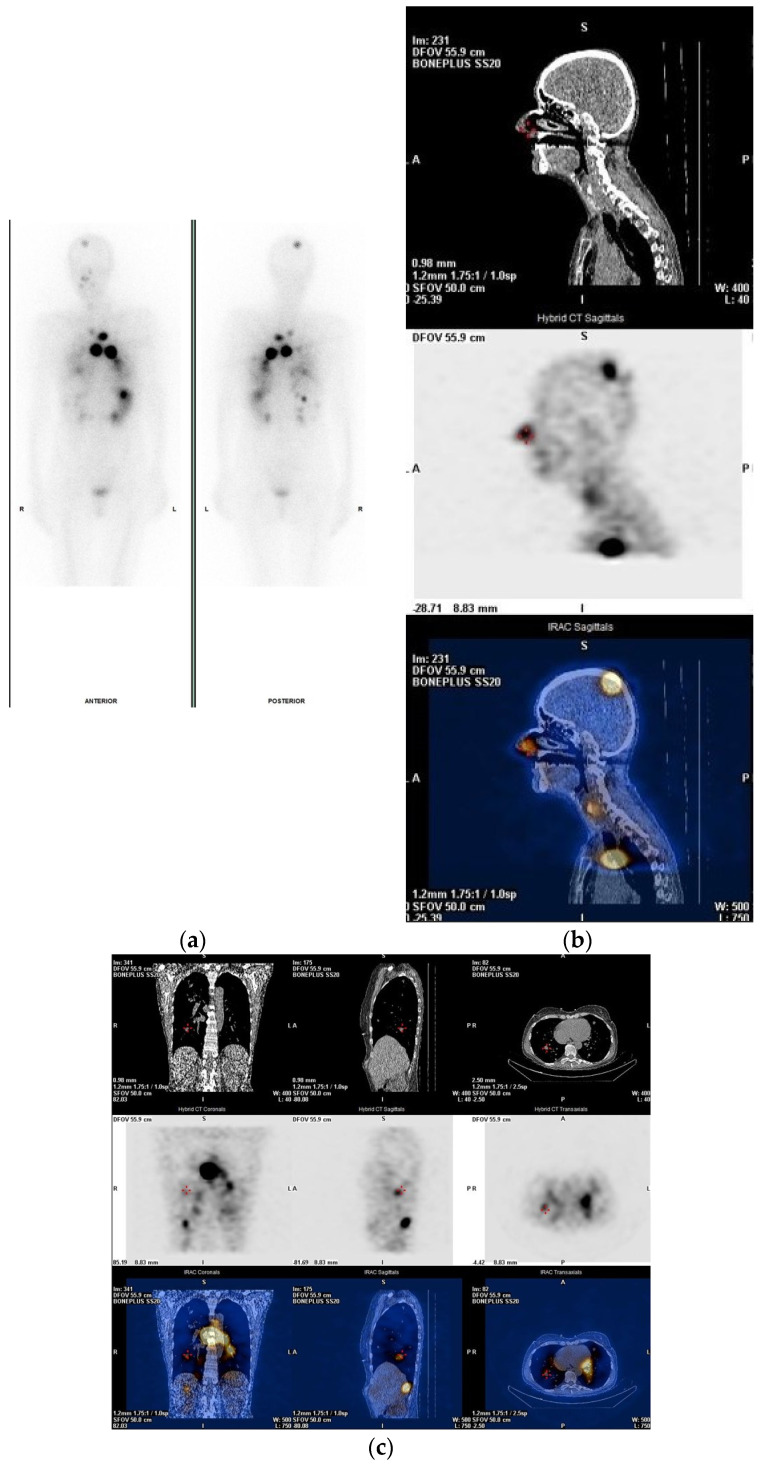

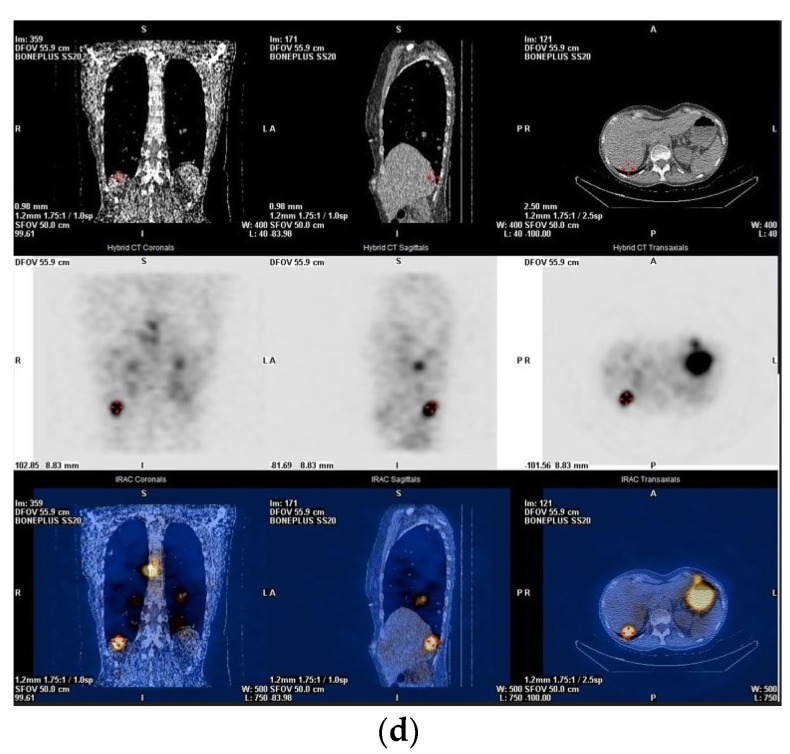
D.B., a 60-year-old woman, after total thyroidectomy and neck dissection, papillary thyroid carcinoma, initially staged pT3bN1M1; previously underwent first radioiodine treatment with 5.55 GBq I-131, then received irradiation of metastatic disease to the right neck and spine VTh5-7, and an additional course of radioiodine therapy of 7.4 GBq (200 mCi) I-131. (**a**) Planar PT-WBI after administration of I-131 of 7.4 GBq (200 mCi) anterior and posterior images, and post-treatment SPECT/CT images. (**b**) Axial CT, SPECT, and SPECT/CT images (**c**) and (**d**) coronal, sagittal and axial planes of CT, SPECT, and SPECT/CT images demonstrate multiple foci localized in the brain, bilateral lungs, mediastinal lymph nodes, and liver corresponding to the metastatic disease.

Post-treatment SPECT/CT imaging (PT-WBS SPECT/CT) increases the diagnostic accuracy of planar whole-body scanning and provides precise anatomic localization and characterization of detected lesions (Figure 5).

Post-treatment scanning with SPECT/CT can detect significantly more neoplastic lesions than planar PT-WB imaging. SPECT/CT scans altered patients’ classification and changed the treatment management in 16% or 25% of cases [55,56], while the therapeutic approach was changed in 41% of patients [57]. Aide et al. reported a difference between post-therapeutic I-131 scans, with 29% indeterminate findings on planar and only 7% on SPECT/CT images, leading to patients’ restaging and expected outcomes during the follow-up [58]. Ciappuccini et al. showed that SPECT/CT imaging integrated with planar WBI has 78% sensitivity and 100% specificity in predicting recurrent or persistent disease [59].

SPECT/CT imaging has an impact on patients’ staging and risk stratification. Malamitsi et al. reported that PT-WBI SPECT/CT was favorable over planar WBI by decreasing false-negative and false-positive findings. In addition, SPECT/CT imaging had a great impact on NM staging compared to the estimated TNM classification based on histopathology and planar WBI. A significant difference was reported in diagnostic accuracy, which was low for planar PT-WBS (51.72%) and very high for PT-WBI SPECT/CT (100%) [60].

In a bi-institutional study, if compared to planar PT-WBI, PT-SPECT/CT WBI altered patients’ risk category in 35.6% of cases, which consequently changed the patient management and follow-up [61].

Several publications have reported the role of post-treatment SPECT/CT in the nodal re-staging of DTC patients. Based on post-treatment SPECT/CT data, in a study by Grewal et al., 15% of post-surgical patients were upstaged from stage N0 to stage N1 [62]. Similar results were reported by Schmidt et al. [63]. In comparison to planar imaging, SPECT/CT downstaged 21% of patients and upstaged 14% of patients. In contrast, based on PT-WBI SPECT/CT images versus PT-WBI scans, Mustafa et al. reported revised nodal stage in as high as 24.5% of patients [64]. In two different studies, Xue et al. [65] and Konfluerst et al. [66] reported the same results, indicating that stage N was altered in 36.4% of patients. The change in N stage consequently altered patient risk stratification in 6.4–25% [65]. In addition, SPECT/CT imaging changed the M stage with upstaging 21.1% of patients from stage M0 to stage M1 [66].

Although pre-treatment and post-treatment SPECT/CT imaging is favorable over planar WB imaging, this technique shows several disadvantages. Additional imaging is time-consuming and causes patient discomfort and risk of claustrophobia in some cases [67]. Misregistration occurs if a patient moves during the acquisition, and the presence of streak artifacts may affect the quality of image interpretation. In addition, radiation exposure from the CT must be considered, especially in pediatric patients [26,68].

## 4. Conclusions

Clinical value of postoperative pre-ablation imaging is limited. Low-dose dx WBI is sub-theranostic due to the radioiodine indifference and low detection sensitivity of the occult cervical metastases. Occult metastases in the neck may only be detected on post-treatment I-131 WBI. PT-WBI has a significant role in the staging of radio-avid DTC patients and detecting non-radio-avid tumors. PT-WBI must be performed after radioiodine treatment in all DTC patients. SPECT/CT improves planar whole-body imaging in both pre- and post-ablation settings. In order to provide the correct initial TNM staging and better risk classification, post-ablation SPECT/CT imaging should be performed in DTC patients whenever possible.

## Data Availability

The data presented in this study are available in the cited References.
